# Supplementary material for: Protocol for the development of a core outcome set for neonatal sepsis (NESCOS)
Source: PLoS One. 2023 Dec 5;18(12):e0295325. doi: 10.1371/journal.pone.0295325 (PMC10697588; doi:10.1371/journal.pone.0295325)
Supplement: S1 File — The complete search strategy of the qualitative systematic review of the NESCOS project. (DOCX) [file pone.0295325.s002.docx]

**NESCOS SEARCH STRATEGY (July 5, 2022)**

|  | Before deduplication | After deduplication |
| --- | --- | --- |
| MEDLINE (Ovid) | 3314 | 2843 |
| EMBASE | 3922 | 2599 |
| CINAHL (EBSCO) | 1350 | 1024 |
| PsycInfo (Ovid) | 408 | 311 |
| **Total** | **8994** | **6777** |

**MEDLINE/OVID: 3314 results**

1. exp Infant/
2. (infan* or newborn or new-born or neonat* or premature or preterm or very low birth weight or low birth weight or VLBW or LBW).mp.
3. 1 or 2
4. exp qualitative research/
5. ((("semi‐structured" or semistructured or unstructured or informal or "in‐depth" or indepth or "face‐to‐face" or structured or guide) adj3 (interview* or discussion* or questionnaire*)) or (focus group* or qualitative or ethnograph* or ethnomethodolog* or "ethnological search" or fieldwork or "field work" or "field study" or "key informant" or phenomenol* or ethnonurs* or "grounded theor*" or "purposive sample" or "theoretical sample" or "cluster sample" or hermeneutic* or heuristic* or semiotics or "lived experience*" or "life experiences" or "thematic analysis" or "discourse analysis" or "constant comparative method")).ti,ab. or interviews as topic/ or focus groups/ or narration/ or qualitative research/
6. 4 or 5
7. (sepsis or septic or sepsisemia or septicemia or bacter* or infect* or (gram adj2 negative)).mp.
8. 3 and 6 and 7

**EMBASE: 3922 results**

1. 'infant'/exp
2. 'infant disease' OR newborn OR neonat* OR premature OR preterm OR 'low birth weight'/exp OR 'birth weight'/exp
3. #1 OR #2
4. 'sepsis'/exp
5. sepsis OR septic OR sepsisemia OR septicemia OR bacter* OR infect* OR (gram AND adj2 AND negative)
6. #4 OR #5
7. 'qualitative research'/exp
8. 'focus group*' OR qualitative* OR ethnograph* OR ethnomethodolog* OR 'ethnological search' OR fieldwork OR 'field work' OR 'field study' OR 'key informant' OR phenomenol* OR ethnonurs* OR 'grounded theor*' OR 'purposive sample' OR 'theoretical sample' OR 'cluster sample' OR hermeneutic* OR heuristic* OR semiotics OR 'lived experience*' OR 'life experiences' OR 'thematic analysis' OR 'discourse analysis' OR 'constant comparative method'
9. ('semi‐structured' OR semistructured OR unstructured OR informal OR 'in‐depth' OR indepth OR 'face‐to‐face' OR structured OR guide) NEAR/3 (interview* OR discussion* OR questionnaire*)
10. #7 OR #8 OR #9
11. #3 AND #6 AND #10

**CINAHL: 1350 results**

1. (MH "Infant+")
2. neo-nat* or neonat* or neo nat* or newborn* or new born* or newly born* or new-born or premature or preterm or very low birth weight or low birth weight or VLBW or LBW
3. S1 OR S2
4. (MH "Sepsis+")
5. sepsis or septic* or infect* or bacter* or sepsisemia or septicemia
6. S4 OR S5
7. (MH "Qualitative Studies+")
8. 'focus group*' OR qualitative* OR ethnograph* OR ethnomethodolog* OR 'ethnological search' OR fieldwork OR 'field work' OR 'field study' OR 'key informant' OR phenomenol* OR ethnonurs* OR 'grounded theor*' OR 'purposive sample' OR 'theoretical sample' OR 'cluster sample' OR hermeneutic* OR heuristic* OR semiotics OR 'lived experience*' OR 'life experiences' OR 'thematic analysis' OR 'discourse analysis' OR 'constant comparative method'
9. ('semi‐structured' OR semistructured OR unstructured OR informal OR 'in‐depth' OR indepth OR 'face‐to‐face' OR structured OR guide) N3 (interview* OR discussion* OR questionnaire*)
10. S7 OR S8 OR S9
11. S3 AND S6 AND S10

**PsycInfo: 408 results**

1. exp Neonatal Period/
2. (infan* or newborn or new-born or neonat* or premature or preterm or very low birth weight or low birth weight or VLBW or LBW).mp. [mp=title, abstract, heading word, table of contents, key concepts, original title, tests & measures, mesh word]
3. 1 or 2
4. exp Qualitative Methods/
5. ('focus group*' or qualitative* or ethnograph* or ethnomethodolog* or 'ethnological search' or fieldwork or 'field work' or 'field study' or 'key informant' or phenomenol* or ethnonurs* or 'grounded theor*' or 'purposive sample' or 'theoretical sample' or 'cluster sample' or hermeneutic* or heuristic* or semiotics or 'lived experience*' or 'life experiences' or 'thematic analysis' or 'discourse analysis' or 'constant comparative method').mp. [mp=title, abstract, heading word, table of contents, key concepts, original title, tests & measures, mesh word]
6. ((semistructured or unstructured or informal or indepth or structured or guide) adj3 (interview* or discussion* or questionnaire*)).mp. [mp=title, abstract, heading word, table of contents, key concepts, original title, tests & measures, mesh word]
7. 4 or 5 or 6
8. (sepsis or septic or sepsisemia or septicemia or bacter* or infect* or (gram adj2 negative)).mp. [mp=title, abstract, heading word, table of contents, key concepts, original title, tests & measures, mesh word]
9. exp Infectious Disorders/
10. 8 or 9
11. 3 and 7 and 10
