# Supplementary material for: Protocol for the development of a core outcome set for neonatal sepsis (NESCOS)
Source: PLoS One. 2023 Dec 5;18(12):e0295325. doi: 10.1371/journal.pone.0295325 (PMC10697588; doi:10.1371/journal.pone.0295325)
Supplement: Attachment — Submitted filename: Response to reviewers.docx [file pone.0295325.s003.docx]

**Response to Reviewers**

1. Please ensure that your manuscript meets PLOS ONE's style requirements, including those for file naming. The PLOS ONE style templates can be found at

https://journals.plos.org/plosone/s/file?id=wjVg/PLOSOne_formatting_sample_main_body.pdf and

https://journals.plos.org/plosone/s/file?id=ba62/PLOSOne_formatting_sample_title_authors_affiliations.pdf

***Changes we made:*** *We carefully reviewed the PLOS ONE style templates and made the necessary adjustments to ensure that our manuscript meets the criteria set by the journal.*

2. Please include captions for your Supporting Information files at the end of your manuscript, and update any in-text citations to match accordingly. Please see our Supporting Information guidelines for more information: http://journals.plos.org/plosone/s/supporting-information.

***Changes we made:*** *We added captions for the Supporting Information file at the end of the manuscript and revised the in-text citations to align with the provided guidelines. We took into account the guidelines while making these updates.*

3. Please review your reference list to ensure that it is complete and correct. If you have cited papers that have been retracted, please include the rationale for doing so in the manuscript text, or remove these references and replace them with relevant current references. Any changes to the reference list should be mentioned in the rebuttal letter that accompanies your revised manuscript. If you need to cite a retracted article, indicate the article’s retracted status in the References list and also include a citation and full reference for the retraction notice.

***Changes we made:*** *We thoroughly reviewed the reference list to confirm its completeness and accuracy. There are no instances of retracted papers within the list. It's important to note that we made no additions or removals of references; our sole focus was on rectifying the reference list itself.*
